# Recent Advancements in 20S Proteasome Enhancement: Degradation of Undruggable Targets

**DOI:** 10.1021/acsmedchemlett.5c00451

**Published:** 2025-09-19

**Authors:** Sydney G. Cobb, Jetze J. Tepe

**Affiliations:** Department of Chemistry, 2358 University of Virginia, Charlottesville, Virginia 22904, United States

**Keywords:** 20S proteasome, protein degradation, proteasome enhancers, intrinsically disordered proteins, neurodegenerative diseases

## Abstract

The ubiquitin-independent proteasome system has emerged as an attractive point of intervention for a variety of diseases, including neurodegenerative diseases. Though inhibition of this system has been studied for decades, 20S proteasome enhancement is much younger by comparison, but substantial levels of progress have been made in this field especially within the last five years. This microperspective will highlight these advancements, focusing on the novel developments being made in designing potent enhancers and evaluating them in disease-relevant systems.

## Graphical abstract

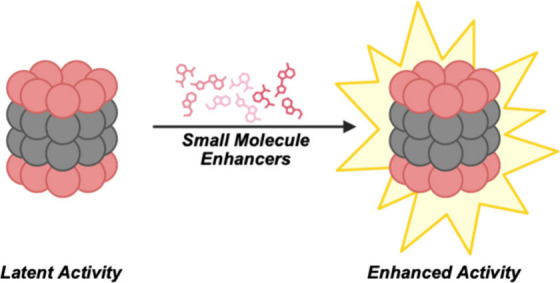

Proteins of diverse sizes and conformations are involved in a host of cellular functions within the body, yet a protein’s lifespan is a highly regulated process that involves synthesis and degradation.^1,2^ The ubiquitin-proteasome system is closely associated with this regulation as a key protein degradation pathway.^3,4^ The 20S core particle of the proteasome is comprised of a stack of four heptameric rings, and its six threonine active sites are housed within the inner two rings, termed the β-rings (Figure 1A).^5,6^ Three catalytic sites are located in each β-ring, and each site possesses a unique selectivity for different amino acid residues. The caspase-like sites are located within the β1-subunits and generally cleave after acidic residues, while the trypsin-like (β2) and chymotrypsin-like (β5) sites cleave after basic and hydrophobic residues, respectively.^5^

**Figure 1:**
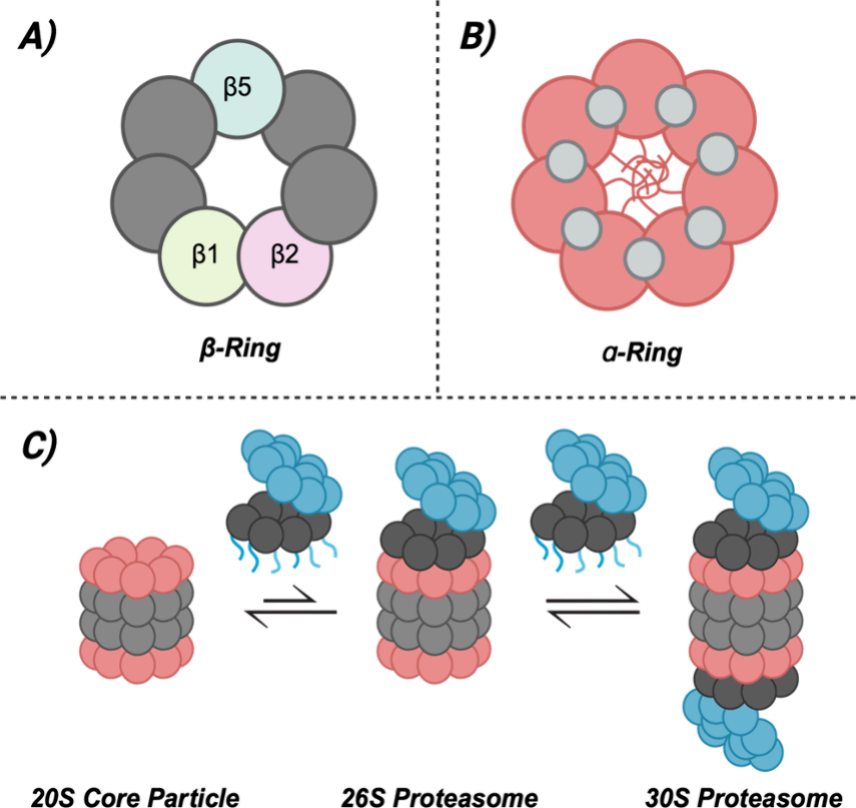
(A) The three different catalytic sites are in the β-rings, with caspase-like activity in the β1 subunit, trypsin-like in β2, and chymotrypsin-like in β5. (B) The α-rings have pockets between each of their subunits and gates at their centers. The intersubunit pockets are pictured in gray, and the gate in this depiction is closed. (C) The different proteasome complexes exist in equilibrium with each other based on the association of the 19S cap.

The outer two rings are known as the α-rings, which each have a small 13 Å pore at their centers ( Figure 1B).^7^ In the 20S’s latent form, the *N*-terminal tails of each α-subunit are extended to cover these gates, thus preventing substrate entry into the catalytic chamber.^8^ Though this closed-gated and inactive form is the latent conformation of the enzyme, the gate oscillates between its closed and open conformation in a 3:1 dynamic equilibrium.^9^ The α-ring surfaces also contain additional pockets between each of their seven subunits, allowing for other enzymatic components to interact with the 20S core particle’s fundamental structure (Figure 1B).^7^ The 19S regulatory particle is one such component and forms the 26S proteasome in an ATP-dependent fashion (Figure 1C).^7,10^ The 30S proteasome is formed when a second 19S cap binds to the other α-ring of the 20S core particle, though this double-capped proteasome is often called the 26S proteasome as well (Figure 1C).^7,10^

Proteasome-mediated proteolysis can occur through two different pathways as dictated by the structure of the protein substrate ( Figure 2). The 26S and 30S proteasomes perform ubiquitin-dependent degradation, which involves a series of ligase-mediated reactions to affix ubiquitin tags on the substrates being degraded.^11^ The 19S cap is necessary for recognizing and removing these ubiquitin tags prior to unfolding and feeding the substrates into the catalytic chamber of the 20S core particle.^12,13^ The 20S core particle on its own can also degrade proteins but only in a ubiquitin-independent manner.^4,14^ Without the 19S cap to identify and unfold its substrates, the 20S core particle is restricted to degrading proteins that are already unfolded.^14^ Recent studies indicate that the interactions between the unfolded substrates and the α-rings facilitate this degradation activity.^15^ The specifics of this are not completely understood, however, and it remains an exciting area for further study.

**Figure 2:**
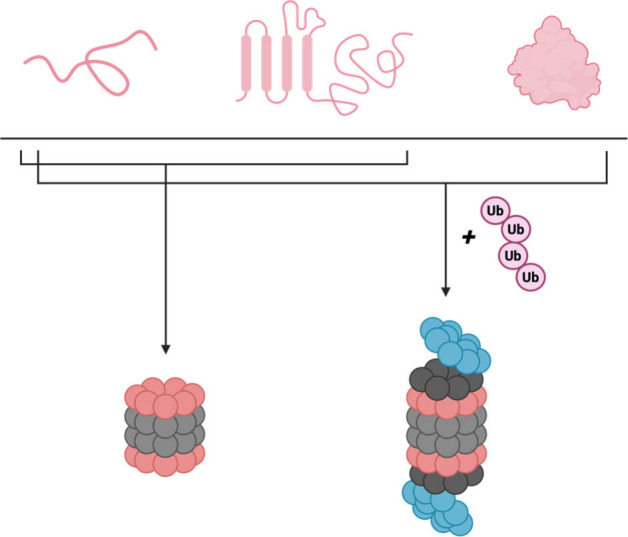
All forms of the proteasome can degrade proteins, but their substrates differ based on protein structure and conformation. The 20S core particle can degrade unfolded proteins (top left protein) or proteins with unfolded regions (top middle protein) without the need for ubiquitination. The 26S/30S proteasomes can degrade both folded (top right protein) and unfolded proteins, but all substrates must be tagged with ubiquitin prior to being degraded.

Intrinsically disordered proteins (IDPs), or proteins that lack a well-defined tertiary structure, encompass a large portion of unfolded proteins and are often substrates of the 20S proteasome.^4,16,17^ Without a stable three-dimensional conformation, IDPs have a flexible and dynamic nature, enabling them to interact with many different binding partners. At the same time, this structural freedom renders IDPs “undruggable” therapeutic targets as they lack defined small molecule binding pockets.^18^ In healthy levels, IDPs are often sequestered in “nanny” complexes to prevent the unfolded proteins from being prematurely degraded and allow them to perform their physiological functions.^19^ Once their purpose has been achieved, IDPs are often rapidly degraded by 20S proteasome or are tagged for degradation by the 26S/30S proteasomes.^19,20^ Despite these regulatory factors, levels of disordered proteins may change for a variety of reasons, including oxidative damage and protein misfolding.^21,22^

As we age, proteasome-mediated proteolysis declines in efficiency.^2^ This phenomenon can be exacerbated to an unhealthy degree especially if it coincides with IDP accumulation. Smith and co-workers elucidated a common mechanism of proteasome impairment by IDPs, finding that smaller soluble oligomers of α-synuclein, amyloid-β, and Htt-53Q were involved in this inhibition.^23^ These soluble oligomers are pathogenic hallmarks of many neurodegenerative diseases, including Parkinson’s disease, Alzheimer’s disease, and Huntington’s disease.^23^ Millions of older adults are affected by these neurodegenerative diseases worldwide, and these numbers are only expected to increase as the global population continues aging.^24^ Given the interplay between neurodegeneration and IDPs, the 20S proteasome has been identified as a promising therapeutic target for disease intervention. While some concerns could be raised about the safety of enhancing the activity of a protein degradation pathway, several gain-of-function studies have determined that increasing proteasome activity often correlates to decreased levels of oxidative stress and increased longevity.^25–27^ Studies have also shown that healthy human centenarians possess sustained levels of proteasome activity.^28^

Extensive research on 20S proteasome enhancement has yielded important insights, uncovering new roles for both large regulatory particles and small molecule enhancers.^29,30^ While the former falls outside the scope of this microperspective, the topic has been recently covered in an elegant and comprehensive review.^31^ Previously discovered small molecule enhancer classes have included imidazolines,^32^ dihydroquinazolines,^33^ substituted indoles,^34^ phenothiazines,^35^ and spirocyclic compounds.^36^ This microperspective will focus on some of the more recent advancements being made in the field of 20S proteasome enhancement, highlighting new developments in identified small molecule enhancers and novel applications of this therapeutic strategy to relevant disease models.

## Novel Enhancer Scaffolds and Therapeutic Applications

Natural products have long been a source of inspiration for drug discovery efforts,^37,38^ so their relevance to the field of 20S proteasome enhancement seems only natural. In fact, betulinic acid, a pentacyclic natural product, was one of the earliest identified enhancers.^39^ Expanding beyond this, the Hitora and Tsukamoto laboratories recently screened a collection of yohimbine-type and ergot alkaloids from the Prestwick Phytochemical Library for 20S proteasome enhancement.^40^ Of the 320 compounds initially tested for chymotrypsin-like activity, only 12 were considered hits after reaching a 4-fold increase in activity at 10 μM. The yohimbine-type alkaloid syrosingopine emerged as their most active hit after producing a 13-fold increase in proteolytic degradation ( Figure 3A), while the other identified hits had activities ranging from four- to nearly 9-fold. These included reserpine (7.9-fold enhancement; another yohimbine-type alkaloid), ursolic acid (6.5-fold; a known 20S proteasome enhancer),^41^ and curcumin (5.4-fold; a presumed PAINS compound).^42^

**Figure 3:**
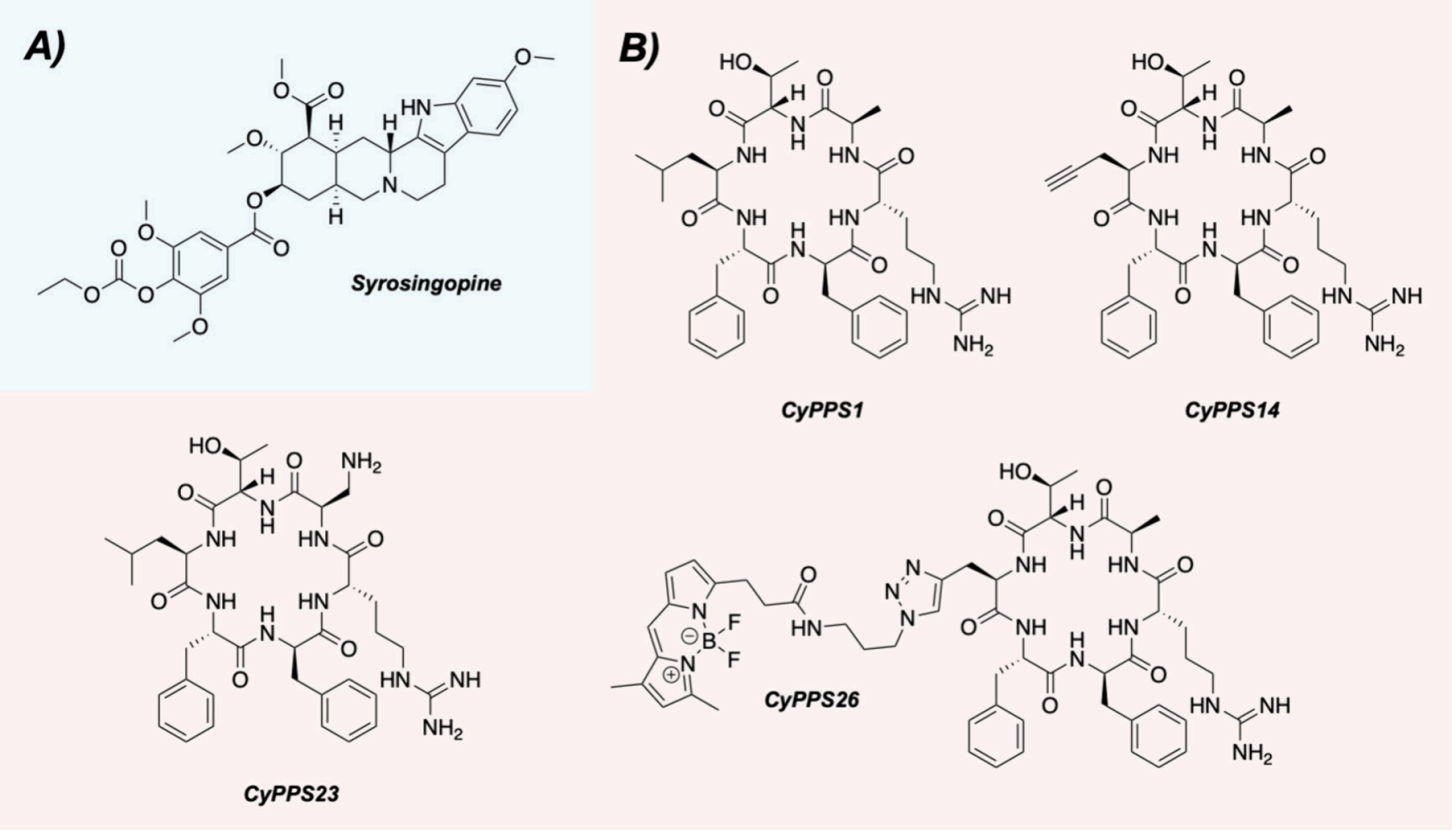
(A) Structure of syrosingopine. (B) Structures of the parent cyclic peptide (**CyPPS1**) and selected analogs.

When evaluated further, syrosingopine and reserpine displayed comparable and potent activities for each of the 20S core particle’s catalytic sites, yet only syrosingopine was able to increase the degradation of α-synuclein. The authors hypothesized this activity difference may result from syrosingopine having a stronger interaction with the 20S proteasome than reserpine, and the nature of this interaction was investigated further in cells. Using a covalent and fluorescence-based proteasome activity probe (Me_4_BodipyFL-Ahx_3_-Leu_3_VS),^43^ the authors explored syrosingopine’s impact on the proteasome in HeLa cells, using chlorpromazine (a known 20S proteasome activator)^35^ and MG-132 (a known 20S proteasome inhibitor)^44^ as controls. After two independent experiments, the samples treated with syrosingopine displayed the most fluorescence, indicating increased levels of proteasome activity. While the authors attribute this result to syrosingopine opening the 20S proteasome gate to allow the probe to bind, additional biophysical experiments could be performed to support this conclusion. Given the complexity of a cellular environment, there’s no guarantee syrosingopine is interacting with the 20S proteasome directly to induce this increase in activity, so the continued investigation of these results remains an intriguing area to explore in future studies.

Derivatives and mimics of natural products can possess potent biological activities as well, making these compounds another interesting area of chemical space to investigate for 20S proteasome enhancement. Building off previous work that developed methods to increase the evaluation of synthetic natural product inspired cyclic peptides (SNaPP),^45^ the Trader and Parkinson laboratories tested previously identified synthetic cyclic peptides for their abilities to enhance the 20S proteasome.^46^

They noted that cyclic peptides offer a host of structural advantages, including increased resistance to degradation, which is a common problem for linear peptides. Starting with 45 cyclic peptides generated by the SNaPP method, these compounds were evaluated in the Trader lab’s previously reported TAS-1 assay,^47^ which utilizes a rhodium-based peptide substrate to monitor 20S proteasome activity over time. Out of their initial 45 compounds, nine were able to increase 20S proteasome activity by at least 20% when evaluated at 10 μM. Selecting the compound with the most activity (**CyPPS1**), they conducted a brief structure–activity relationship (SAR) study to evaluate the importance of each amino acid in the scaffold and whether the overall activity could be improved ( Figure 3B). A handful of their 37 analogs possessed either similar or increased activities, including **CyPPS14** and **CyPPS23.**

The continued evaluation of these new analogs yielded interesting results. In an eight-point dose response, all compounds plateaued at higher concentrations, yet the maximal responses (reported *E*_max_ values) varied from 283% to 701%. As a result, the reported EC_50_ values were also quite different. In fact, their most active compounds in terms of their EC_50_ values were often not the ones with the highest *E*_max_ values, meaning their most potent compounds were not simultaneously producing the greatest enhancing effects on the 20S proteasome’s overall activity. While an interesting dilemma, such a result may not be surprising as different compounds may enhance the 20S proteasome in different ways. For instance, the activity of one may disproportionately enhance caspase-like activity over the other two sites while another compound may favor enhancing trypsin-like activity. The three sites may not have equal thresholds for maximal activity either, so enhancing the caspase-like site more than the other two may have variable consequences for the maximal response of the 20S proteasome overall. Having a deeper understanding of how the 20S core particle’s maximal response may relate to a compound’s overall efficacy remains a significant area for further exploration within the field.

The same analogs were also evaluated for their abilities to impact the levels of proteins with ranging levels of structural order. When investigating α-synuclein (disordered), GAPDH (ordered), and lysozyme (ordered), their lead compound (**CyPPS1**) and two analogs (**CyPPS14** and **CyPPS23**) demonstrated the ability to enhance the degradation of α-synuclein without impacting the levels of the two ordered proteins. As these three compounds were moved into cellular assays, their initial studies evaluated whether the cyclic peptides would be cell permeable. Using a BODIPY-labeled analog (**CyPPS26**) in A549 cells, they were able to visualize the movement of their compound using confocal microscopy and propose that the mechanism of permeability was likely endocytosis. With some certainty behind the cellular permeability, the authors were able to demonstrate that their compounds could enhance the 20S proteasome in HEK293T cells.

Though linear peptides are “vulnerable” 20S proteasome enhancers as they’re susceptible to being degraded, many of these linear scaffolds do possess the ability to enhance the proteolytic activity of the 20S proteasome. Such peptide activators are often designed to mimic the binding tails of regulatory particles or other caps.^48,49^ The Jankowska group has taken a similar approach in using sequences from the C-terminus of the Blm-10 yeast activator,^50^ which the authors noted conserves the known HbYX sequence that drives peptide tail binding and activity. Starting from their previously published Blm-pep scaffold,^51^ they undertook an SAR study guided by molecular modeling to generate a small library of new peptides ( Table 1). When looking at each catalytic site individually, many of their analogs aligned themselves with the trends of the parent scaffold, which appeared more active for the caspase-like and chymotrypsin-like sites. A notable exception to this was compound **7**, which displayed the highest trypsin-like activity out of the 25 synthesized analogs by far. The authors validated these results using a longer FRET-based DabEDS probe,^41^ which is less prone to digestion by the latent 20S core particle and requires more than a single active site for its degradation. Eight of their compounds were tested alongside Blm-pep, finding that four of them (compounds **2**, **14**, **17**, and **18**) increased the degradation of the DabEDS probe to a greater extent than Blm-pep.

**Table 1:** Sequences of Select Blm-pep Analogs^a^

position	**1**	**2**	**3**	**4**	**5**	**6**	**7**	**8**	**9**	**10**	**11**	**12**	**13**	**14**
Blm-pep	K	Y	F	T	G	S	K	L	W	R	S	Y	Y	A
2	K		F	T	**Q**	**K**	**P**	L	W	R	S	Y	Y	A
7	**E**		F	T	**D**	**E**	**P**	L	W	R	S	Y	Y	A
14	K	Y	F	T	G	S	K	**D**	**Y**	R	**R**	Y	Y	**S**
17	K	Y	F	T	G	S	K	**E**	W	R	S	Y	Y	**T**
18	K	Y	F	T	G	S	K	**D**	W	R	S	Y	Y	**S**

a Analog-specific sequence modifications are highlighted by bold letters.

Noting that their linear peptides may undergo degradation by the 20S core particle, the authors investigated the potential that their peptide analogs may be substrates themselves. Interestingly, their active analogs were all substrates to some degree while their inactive compound went virtually untouched. Attributing this as potential evidence for an allosteric mechanism, they asserted their analogs may loosen the closed-gate conformation of the proteasome to promote the degradation of the probe substrates and peptide enhancers alike. This does resemble the proposed mechanism of 20S proteasome-mediated IDP degradation,^15^ yet the vulnerability of their peptide substrates could still be a barrier to their therapeutic development.

When evaluated in HEK293T cells, compounds **17** and **18** proved effective in impacting the levels of transiently transfected GFP-tau and GFP-SOD1 (SOD1G37R) when treated at the time of transfection. Interestingly, degradation of both proteins was reduced when higher concentrations of peptide analogs were used. After ruling out potential interactions with the 26S proteasome in a purified assay and compound self-aggregation, the authors hypothesized that their peptides may either bind with the transfected protein constructs to prevent their degradation or impact additional cellular pathways in ways that impair the 20S proteasome at higher concentrations. To determine that their compounds do interact with the 20S proteasome, though, the authors used X-ray crystallography techniques to show that compound **18** binds in three of the intersubunit pockets on the α-ring. Despite being present in several pockets, this did not lead to an open-gated conformation of the 20S proteasome. Instead, their compound impacted the S1 pocket conformations in the three catalytic sites, supporting their hypothesis of an allosteric mechanism of action.

Additional biophysical experiments focused on understanding the binding sites of 20S proteasome enhancers have been recently conducted by Coletta and co-workers using a tetra-anionic porphyrin compound ( Figure 4A).^52^ The work builds off previous studies examining cationic porphyrin scaffolds,^53^ which have been proposed to function as tunable “electrostatic key codes.” Despite having its electronics flipped, this new tetra-anionic scaffold (**H2TPPS**) enhanced the chymotrypsin-like activity of the 20S proteasome in purified assay conditions.

**Figure 4:**
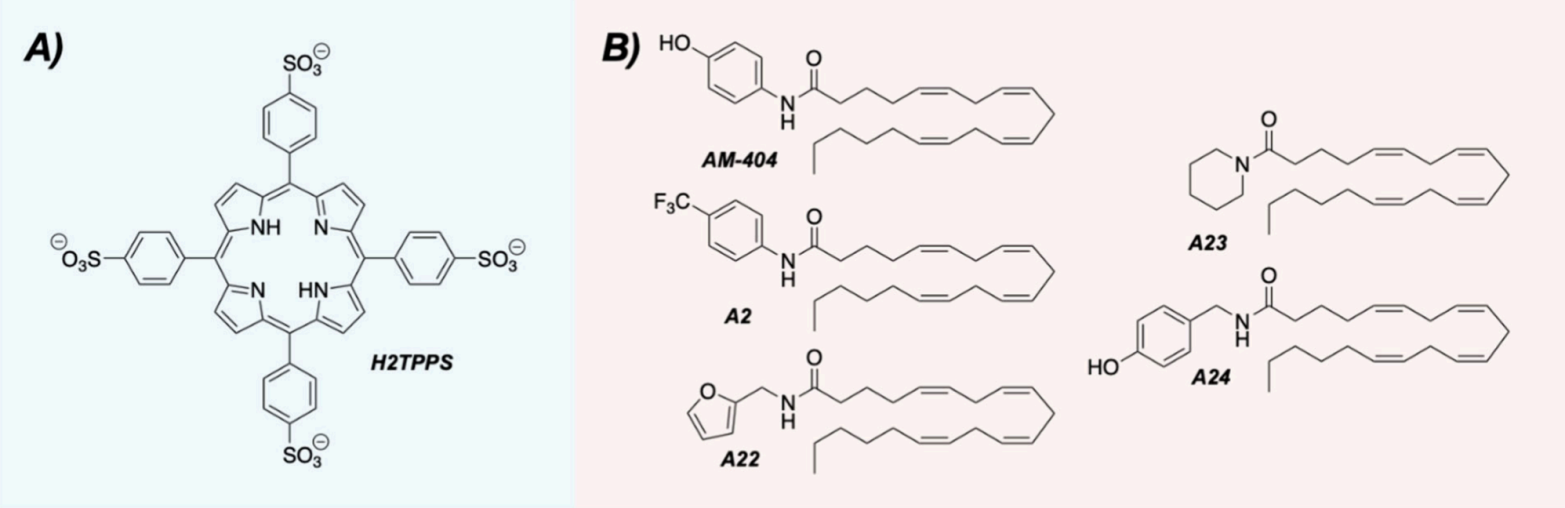
(A) Structure of **H2TPPS**. (B) Structures of the parent compound (**AM-404**) and select analogs.

The authors then turned their attention to atomic force microscopy (AFM) imaging studies to determine how their compound may impact the gating states of the 20S proteasome. By categorizing the particles into “open,” “intermediate,” or “closed” states, they observed the effects of adding a peptide activity probe, Suc-LLVY-AMC, in 20S proteasome samples both with and without their compound. In their untreated control, the closed-gate state predominated. In the presence of 100 μM of the peptide probe, these conformational ratios quickly shifted to favor more intermediate and open-gated states. **H2TPPS** also induced these changes, though the effects occurred more slowly by comparison. Encouraged by these results, the authors performed dynamic molecular docking studies to determine potential locations of compound binding. Based on their outlined protocol and docking results, the authors hypothesized **H2TTPS** may bind to regions near the α1-α2 and α3-α4 grooves in locations that mimic and overlap with the known binding interactions of regulatory particles.

While the peptide HbYX motif is important for activity, nonpeptide small molecules can still induce 20S proteasome activity in the absence of this structural feature. **AM-404**, an arylated arachidonic acid derivative discovered by the Kodadek lab,^34^ is among these compounds with a published EC_50_ value of 28 μM.^54^ While a previous report determined the fatty acid side chain was necessary for activity, the polar aryl-amide headpiece was unexplored at the time.^54^ In this elegant second-generation SAR study, a variety of aromatic and aliphatic ring systems were incorporated in its place (Figure 4B).^55^ Unlike the previous study which failed to identify a more active analog,^54^ several of the new compounds were found to enhance the 20S proteasome more potently than **AM-404** in their published rhodamine-based assay.^47^ Selecting the four with the highest levels of activity (**A2**, **A22**, **A23**, and **A24**), they next explored whether the effects would translate to cell culture. Using the cellular BODIPY-based proteasome activity probe (Me_4_BodipyFL-Ahx_3_-Leu_3_VS), two of their analogs (**A22** and **A23**) displayed more proteasome enhancement than **AM-404** in HEK293T cells.

Given **AM-404**’s history as being somewhat cytotoxic,^54^ complementary cell viability assays were performed to determine whether their new enhancers had mitigated this effect. When tested in HEK293T cells in concentration ranges starting at 100 μM, the analogs did show a reduction in toxicity as compared to **AM-404** after 24 h. Finally, to determine whether these improvements would translate to the degradation of full-length proteins, **A22** and **A23** were evaluated for their abilities to impact the degradation of disordered proteins. Both analogs were able to reduce the levels of disease-associated proteins, including tau-F and α-synuclein, without impacting the levels of structured proteins. This increased activity extended to restoring impaired 20S proteasome activity. When the 20S proteasome was pretreated with inhibitory α-synuclein oligomers, **A22** could restore its proteolytic activity to basal levels while **A23** was able to enhance activity by an additional 50% at 25 μM.

Our lab has done extensive work in developing and identifying novel small molecule enhancers of the 20S proteasome,^33,35,36,56,57^ and a recent study revealed the discovery of potent third-generation chlorpromazine analogs for use in multiple system atrophy (MSA) systems.^58^ Much like Parkinson’s disease, MSA is characterized by the accumulation and aggregation of α-synuclein,^59^ but this protein aggregation is often accompanied by the colocalization of p25α, another disordered protein, within the protein aggregates.^60^ This presented an opportunity to investigate whether 20S proteasome enhancement could interrupt the pathology of MSA through the accelerated degradation of both IDPs. Before exploring this, a brief SAR was conducted to improve the compound class’s translatability to cellular conditions. Previous work with this series demonstrated potent *in vitro* activities that were less impactful in cells,^56^ which was attributed to the phenothiazine core’s potential for being promiscuous and susceptible to metabolism. Alternative heterocycles were incorporated in its place to enhance the *in vitro* potency, leading to the development of two novel carbazole-based compounds, **1** and **4** (Figure 5A). Unlike the previous generation of chlorpromazine analogs, the new analogs’ potencies were well preserved in cells. After transiently transfecting HEK293T cells to express the A53T mutant of α-synuclein, both analogs were able to reduce the mutant protein levels when submicromolar concentrations of each were used.

**Figure 5:**
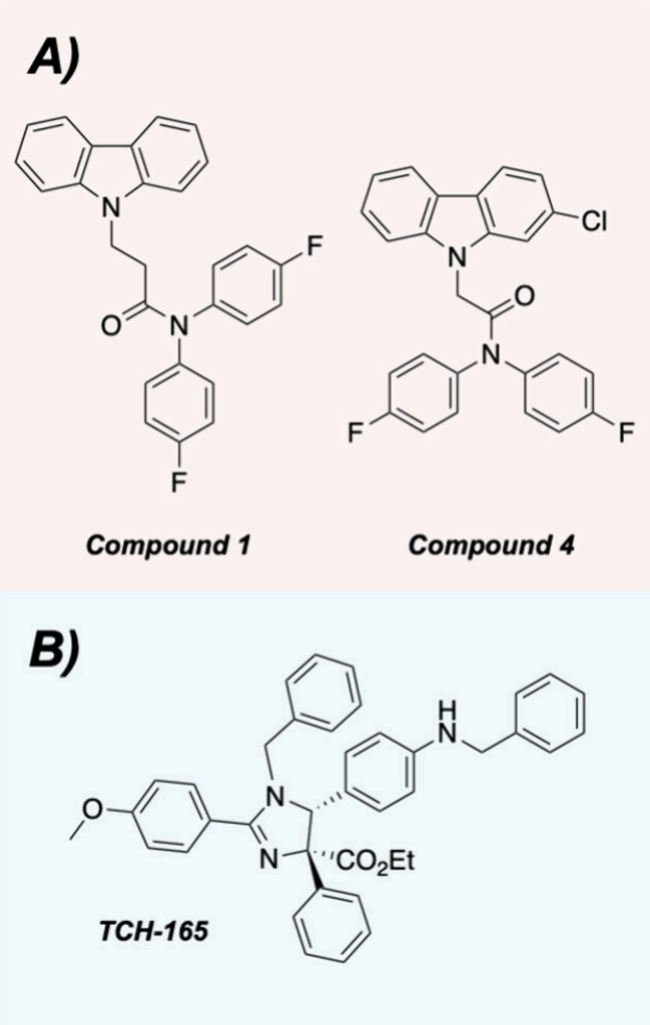
(A) Structures of the improved carbazole analogs. (B) Structure of TCH-165.

While the 20S proteasome-mediated degradation of α-synuclein is known, it was unknown whether the ubiquitin-independent degradation system was involved in the clearance of p25α. We identified that p25α is also a substrate of the 20S proteasome in purified conditions and that both compounds could increase its degradation. This effect translated to cellular environments, where both compounds reduced the levels of transiently transfected p25α in PC12 cells. Given the coaggregative relationship between α-synuclein and p25α in MSA, we were excited to see that 20S proteasome enhancement could impact the levels of both disease-relevant proteins individually. From there, we hypothesized that our small molecule enhancers could assist in preventing the coaggregation and seeding events in cells. It has been well documented that proteopathic seeds can spread in a prion-like manner and may drive the induction and progression of neurodegenerative diseases, including synucleinopathies and tauopathies.^61^ To explore this, we shifted focus toward recapitulating the protein colocalization events. Using PC12 cells that were transiently transfected with the same A53T α-synuclein mutant, MSA-like protein aggregation was induced upon adding p25α protein directly to the media. Using the fluorescent thioflavin-like RB1 probe to assess the degree of aggregation,^62^ both compounds sharply reduced the levels of aggregation relative to the untreated control. This extended to 20S proteasome impairment studies where the coaggregation of A53T α-synuclein and p25α caused a decrease in 20S proteasome activity that compounds **1** and **4** were able to restore.

Our lab has also recently investigated the applicability of 20S proteasome enhancement to amyotrophic lateral sclerosis (ALS) disease models.^63^ Familial ALS pathology is often characterized by motor neuron degeneration and the accumulation of several dipeptide repeat (DPR) proteins.^64–66^ The most toxic of these DPR proteins are arginine-rich and consist of poly-GR and poly-PR repeats.^67,68^ Taking into consideration the presence of basic residues in the most toxic DPR proteins and their presumed identity as IDPs,^69^ we hypothesized that 20S proteasome enhancement could be used as a therapeutic strategy for impacting ALS disease models. Proof-of-concept studies using TCH-165 (a known 20S proteasome enhancer; Figure 5B)^32^ and a short fluorogenic poly-GR_3_ probe revealed enhanced and potent degradation of the arginine-rich peptide. When incubated with a longer HA-tagged version of the probe, TCH-165 caused a dose-dependent decrease of an HA-GR_20_ substrate after 30 min of incubation. This effect was also seen with a complementary HA-PR_20_ probe, though its degradation was less pronounced after incubation for 8 h. This difference was largely attributed to the inflexible structural constraints of the prolines in the poly-PR substrate.

The poly-GR and poly-PR substrates were also evaluated for their potential to contribute to 20S core particle impairment. In the presence of the DPR substrates alone, the 20S proteasome proved less efficient in degrading the fluorogenic Suc-LLVY-AMC activity probe. Mitigation of this impairment was then evaluated using TCH-165 to gauge the feasibility of both preventing and restoring impaired proteolytic activity. When TCH-165 was added before the poly-DPR proteins, increasing concentrations of the enhancer revealed increasing levels of 20S activity, which successfully prevented IDP-mediated proteasome impairment. As for restoring function when pretreated with the poly-DPR proteins, the results mirrored the previous protein degradation assays. TCH-165 was only able to restore function with the poly-GR substrate, which was consistent with the enhancer’s reduced ability to enhance the degradation of the poly-PR substrate.

To determine whether these protective effects would translate to a more physiologically relevant system, rat cortical neurons were infected with HSV-GFP-GR_50_ and HSV-GFP-PR_50_ vectors prior to treatment with TCH-165. After 48 h, TCH-165 was able to reduce the accumulation of GR_50_-GFP but did not impact the levels of PR_50_-GFP. Despite being unable to increase the degradation of the PR_50_-GFP substrate levels, treatment with TCH-165 did restore balance to the overall ubiquitin-proteasome system as monitored by total ubiquitin levels, and this effect was observed for both DPR substrates. When these studies were further extended to neuronal survival studies using the GR_50_-GFP and PR_50_-GFP substrates, similar effects were observed. When infected with both arginine-rich substrate vectors alone, fewer rat neurons survived after a five-day period. In the same time frame, more neurons survived if treated with TCH-165 after vector infection, demonstrating a neuroprotective effect.

## Conclusions and Future Perspectives

Though long considered an inactive enzyme altogether, the 20S proteasome remains an important aspect of the ubiquitin-proteasome degradation system as it assists in maintaining levels of intrinsically disordered and other unfolded proteins through a ubiquitin-independent process.^4^ During aging, the 20S proteasome’s ability to degrade these proteins often declines, making it vulnerable to changes in unfolded protein levels.^2^ Such changes can occur in neurodegenerative diseases, which are commonly associated with the aberrant accumulation of IDPs.^23^ For these reasons, targeting the 20S proteasome with small molecule enhancers has recently emerged as a potential therapeutic strategy for overcoming such protein accumulation.

Though years of work has been done in this field, 20S proteasome enhancement remains in its early stages. Several diverse classes of small molecules have demonstrated an ability to enhance the degradation of disease-relevant proteins,^70^ yet the mechanism of action behind these changes remains elusive. Unlike the work of 20S proteasome inhibition, which generally targets the enzyme’s active sites to prevent protein degradation, the location of binding for most 20S proteasome enhancers is largely unclear. Several laboratories, including ours, hypothesize that these enhancers may bind in the intersubunit pockets on the α-ring surfaces. While X-ray crystallography studies using the Blm-pep analogs did find evidence of this,^50^ it is unclear whether this phenomenon will extend to all classes of enhancers, thus cementing a unified mechanism of enhancement, or if different structural classes may bind in slightly different locations and ways. If different compound classes do bind in different locations, this may have unique consequences on the mechanism of 20S proteasome gate opening and the activities of each catalytic site. Studies using AFM imaging have noted that compound treated samples can induce an increase in the number of open-gated 20S proteasomes, demonstrating a shift in the 20S proteasome gating equilibrium.^32,52^ However, conclusive structural data proving that these small molecule enhancers bind to the surface of the α-ring to induce this gating change has yet to be obtained.

Outside of these mechanistic considerations, additional questions remain when evaluating the therapeutic efficiency of 20S proteasome enhancers. While several assays have been established to evaluate the activity of the 20S proteasome, the degree to which the small molecules enhance the enzyme in these assays will often vary from class to class, and rationalizing why this may be is especially challenging given the obscurity in the mechanism of action. For example, two compounds at the same concentration may have vastly different responses. Supposing one yields a 30% increase in activity while the other increases it by 5-fold, is the activity of the first compound really enough to call it an enhancer? Conversely, is the activity of the second too extreme, thus rendering the system vulnerable to enhanced degradation of off-target proteins? For these reasons, additional work investigating how much enhancement is needed to deliver a therapeutic effect and what the widespread effects of 20S proteasome enhancement are will be of the utmost importance prior to its more in-depth clinical evaluation. As these studies are continuing, however, the field of 20S proteasome enhancement remains one of significant therapeutic potential, especially for diseases that currently have no cures. **Safety**. No unexpected or unusually high safety hazards were encountered.

## Data Availability

The data underlying this study are available in the published article.

## Abbreviations

AFM: atomic force microscopy
ALS: amyotrophic lateral sclerosis
ATP: adenosine triphosphate
BODIPY: boron-dipyrromethane
DPR: dipeptide repeat proteins
EC_50_: half-maximal effective concentration
*E*_max_: maximal response
FRET: fluorescence resonant energy transfer
GAPDH: glyceraldehyde-3-phosphate dehydrogenase
GFP: green fluorescent protein
GR: glycine–arginine
HA: hemagglutinin
HbYX motif: sequence of a hydrophobic amino acid, a tyrosine, then any amino acid
HSV: herpes simplex virus
Htt-53Q: Huntington protein construct, with 53 polyglutamine repeats
IDP: intrinsically disordered protein
MSA: multiple system atrophy
PAINS: pan-assay interfering compounds
PR: proline–arginine
SAR: structure–activity relationship
SNaPP: synthetic natural product inspired cyclic peptides
SOD1: superoxide dismutase type 1
Suc-LLVY-AMC: succinyl-leucyl-leucyl-valyl-tyrosyl-7-amido-4-methylcoumarin
Ub: ubiquitin
